# *Bifidobacterium longum* Alleviates Dextran Sulfate Sodium-Induced Colitis by Suppressing IL-17A Response: Involvement of Intestinal Epithelial Costimulatory Molecules

**DOI:** 10.1371/journal.pone.0079735

**Published:** 2013-11-08

**Authors:** Eiji Miyauchi, Tasuku Ogita, Junki Miyamoto, Seiji Kawamoto, Hidetoshi Morita, Hiroshi Ohno, Takuya Suzuki, Soichi Tanabe

**Affiliations:** 1 Graduate School of Biosphere Science, Hiroshima University, Higashi-Hiroshima, Hiroshima, Japan; 2 Laboratory for Intestinal Ecosystem, RIKEN Center for Integrative Medical Sciences (IMS-RCAI), Yokohama, Kanagawa, Japan; 3 Graduate School of Advanced Sciences of Matter, Hiroshima University, Higashi-Hiroshima, Hiroshima, Japan; 4 School of Veterinary Medicine, Azabu University, Sagamihara, Kanagawa, Japan; Virginia Tech, United States of America

## Abstract

Although some bacterial strains show potential to prevent colitis, their mechanisms are not fully understood. Here, we investigated the anti-colitic mechanisms of *Bifidobacterium longum* subsp. *infantis* JCM 1222^T^, focusing on the relationship between interleukin (IL)-17A secreting CD4^+^ T cells and intestinal epithelial costimulatory molecules in mice. Oral administration of JCM 1222^T^ to mice alleviated dextran sulfate sodium (DSS)-induced acute colitis. The expression of type 1 helper T (Th1)- and IL-17 producing helper T (Th17)-specific cytokines and transcriptional factors was suppressed by JCM 1222^T^ treatment. Intestinal epithelial cells (IECs) from colitic mice induced IL-17A production from CD4^+^ T cells in a cell-cell contact-dependent manner, and this was suppressed by oral treatment with JCM 1222^T^. Using blocking antibodies for costimulatory molecules, we revealed that epithelial costimulatory molecules including CD80 and CD40, which were highly expressed in IECs from colitic mice, were involved in IEC-induced IL-17A response. Treatment of mice and intestinal epithelial cell line Colon-26 cells with JCM 1222^T^ decreased the expression of CD80 and CD40. Collectively, these data indicate that JCM 1222^T^ negatively regulate epithelial costimulatory molecules, and this effect might be attributed, at least in part, to suppression of IL-17A in DSS-induced colitis.

## Introduction

Inflammatory bowel diseases (IBD) such as ulcerative colitis and Crohn’s disease are chronic inflammatory disorders of the gastrointestinal tract. Although their etiology is not fully understood, an abnormal immune response against luminal antigens including commensal bacteria is considered the main pathogenesis of IBD [1]. A layer of intestinal epithelial cells (IECs) physically separates mucosal immune cells from luminal antigens and plays a key role in maintaining intestinal immune homeostasis.

Recent studies demonstrated that IECs could function as antigen-presenting cells [2-4]. IECs constitutively or inductively express molecules required for antigen presentation to T cells, including MHC molecules and costimulatory molecules. Furthermore, IECs from IBD patients express elevated levels of these molecules [4-6], implying that inflamed IECs might be involved in excessive activation of the immune system and disturbed intestinal immune homeostasis. Indeed, IECs from IBD patients induced CD4^+^ T cells to proliferate and produce interferon (IFN)-γ in a cell-cell contact-dependent manner [4].

Increasing evidence suggests that type 1 helper T (Th1) and interleukin (IL)-17 producing helper T (Th17) cells are important mediators of inflammation in IBD [7,8]. Th1- and Th17-type cytokines are highly expressed in inflamed mucosa in IBD patients, and inhibition of these cytokines leads to remission of human IBD and murine dextran sulfate sodium (DSS)-induced colitis model [9-13]. The involvement of Th17 cells in the pathogenesis of IBD is also supported by recent genome-wide association studies, indicating that genes involved in Th17 differentiation are associated with susceptibility to IBD [14].

We and other investigators have shown that some commensal and probiotic bacteria alleviate inflammation in human IBD and murine colitis models [15-19]. Several mechanisms are indicated for their beneficial effects including competitive inhibition of pathogen adherence to IEC [20], maintenance of intestinal barrier function [16,21], and the induction of anti-inflammatory immune responses [17,18]. We previously reported that *Bifidobacterium longum* subsp. *infantis* JCM 1222^T^ (type strain) suppressed IL-17A production in a mouse colon organ culture [22]. Thus, this strain might improve intestinal inflammation, at least partially, by the suppression of IL-17A secreting CD4^+^ T cells.

The aim of the present study was to investigate the anti-inflammatory effects and mechanisms of JCM 1222^T^ on DSS-induced acute colitis in mice. We show here that JCM 1222^T^ negatively regulates the expression of intestinal epithelial costimulatory molecules, resulting in the suppression of IL-17A response and colitis.

## Results

### *B. longum* subsp. *infantis* JCM 1222^T^ suppresses DSS-induced colitis and colonic cytokine production

**Figure 1:**
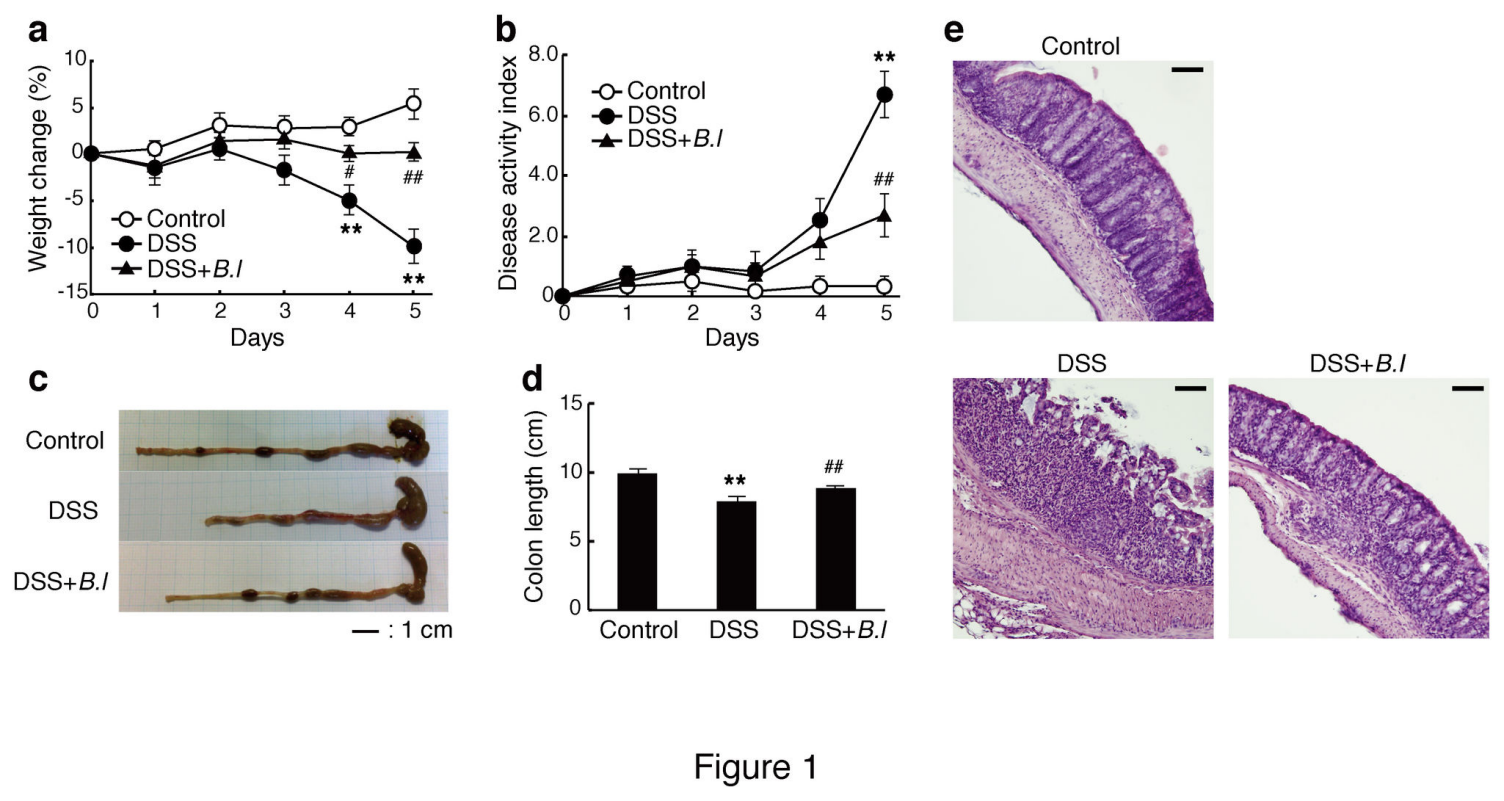
Oral treatment of *B. longum* JCM 1222^T^ (B.l) alleviates DSS-induced acute colitis. Mice were monitored daily for weight loss (a) and DAI (b). On day 5, the entire colon was removed (c), and the length was measured (d). The data are representative of four experiments. Colonic tissue sections were stained with hematoxylin-eosin for histological examination (e). The data are representative of two experiments. Scale bars represent 100 μm. The data are Results are expressed as means ± standard error (n = 4). ^**^ *p*<0.01 versus control mice (Control); ^#^ *p*<0.05 and ^##^ *p*<0.01 versus DSS-treated mice (DSS).

**Figure 2:**
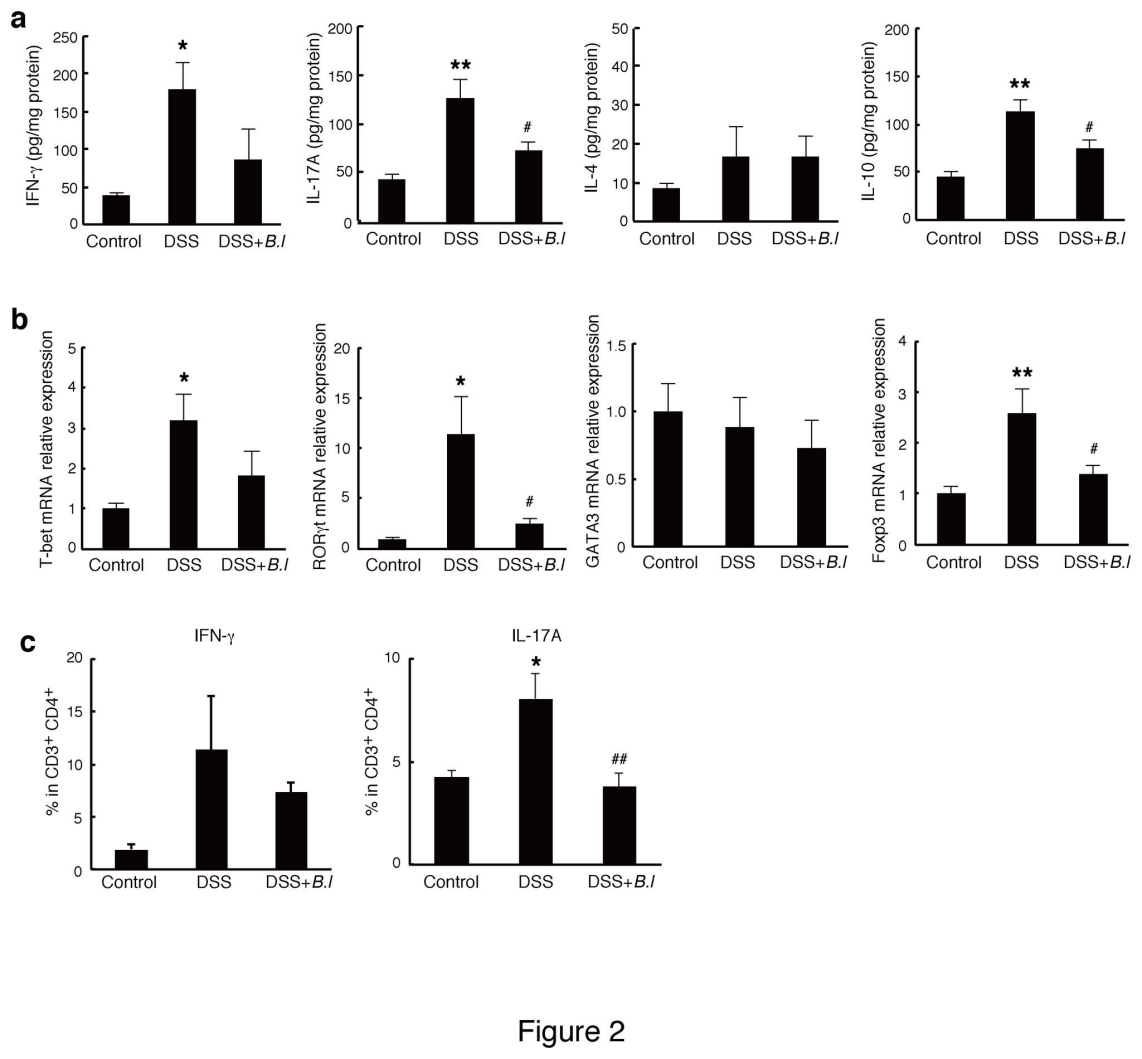
Oral treatment of *B. longum* JCM 1222^T^ (*B.l*) suppresses Th17-specific cytokines and transcription factors. (a) Cytokine production from colonic tissue culture was measured by ELISA. (b) mRNA expression of transcription factors in LPL was analyzed by quantitative PCR. Levels of mRNA were normalized to β-actin mRNA, and expressed relative to control mice. (c) LPL was analyzed for cytokine-expression profiles by intracellular cytokine staining. The frequency of CD4^+^ T cells expressing the indicated cytokines is shown (n = 3-6). The data are representative of two experiments. Results are expressed as means ± standard error (n = 4). ^*^ *p*<0.05 and ^**^ *p*<0.01 versus control mice (Control); ^#^ *p*<0.05 versus DSS-treated mice (DSS).

We first investigated whether oral administration of JCM 1222^T^ suppressed DSS-induced acute colitis in mice. The daily administration of JCM 1222^T^ was effective in preventing body weight loss (Figure 1a), improving Disease Activity Index (DAI) (Figure 1b), and maintenance of colon length (Figure 1c,d). Histological assessment confirmed that DSS treatment induced marked destruction of epithelial cells with crypt loss and hypertrophy of mucosa, whereas the degree of tissue damage in JCM 1222^T^-treated mice was attenuated (Figure 1e). Colitic mice showed a high expression of pro-inflammatory cytokines IFN-γ and IL-17A, as well as anti-inflammatory cytokine IL-10 in the colon, whereas JCM 1222^T^ attenuated the production of these cytokines (Figure 2a). The mRNA expression of T-bet, RORγt, and Foxp3 (Th1-, Th17-, and regulatory T cell-specific transcriptional factor, respectively) were also decreased by JCM 1222^T^ treatment (Figure 2b). Th2-associated cytokine and transcription factor (IL-4 and GATA3) were not affected. Flow cytometric analysis revealed that the population of IL-17A secreting CD4^+^ T cells was significantly decreased by JCM 1222^T^ treatment (Figure 2c). The administration of JCM 1222^T^ alone in control mice did not affect these observations (data not shown).

### Inflamed IEC, but not JCM 1222^T^-treated IEC, induce IL-17A responses

**Figure 3:**
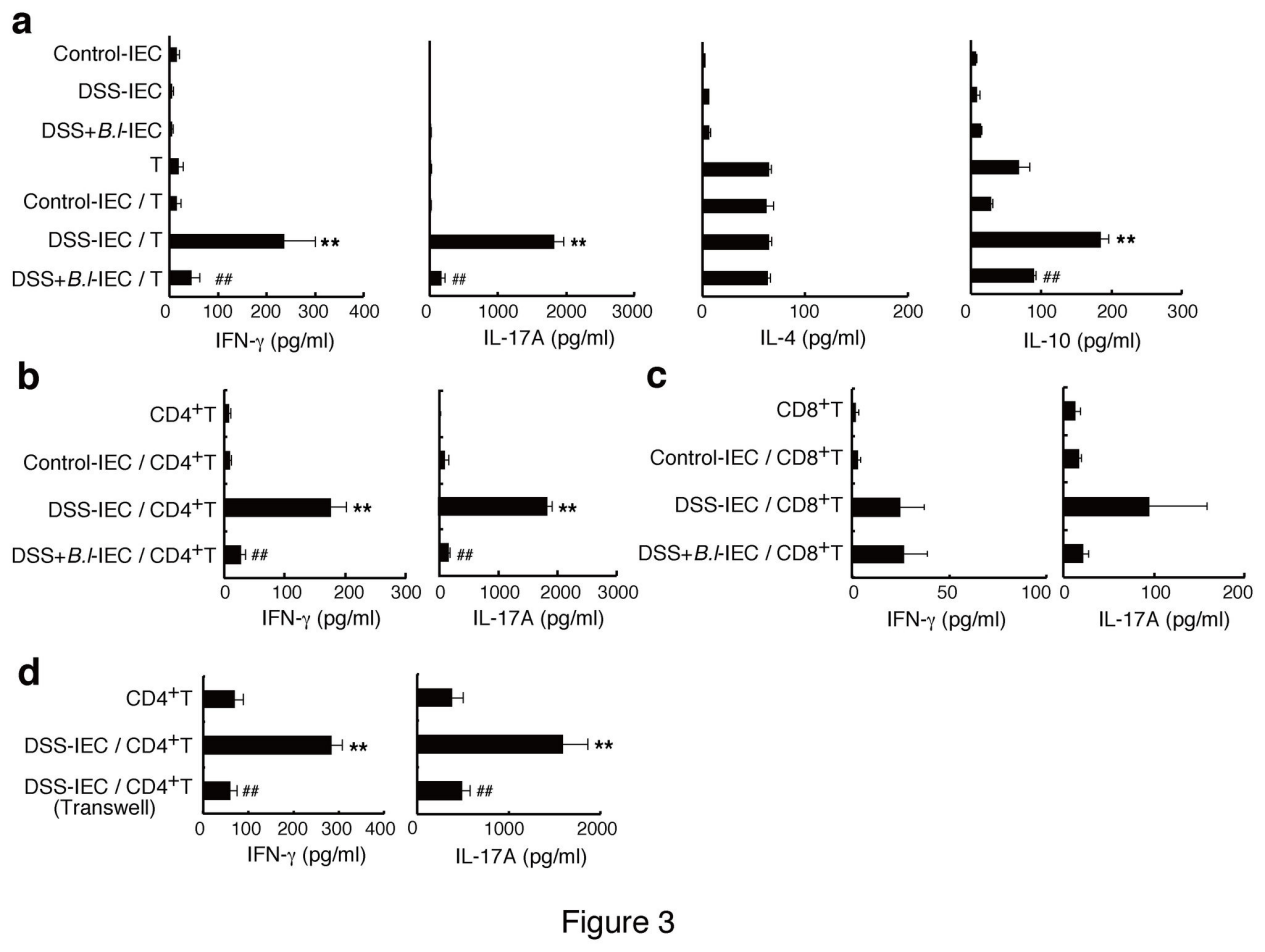
IECs from DSS mice, but not *B. longum* JCM 1222^T^ (B.l) -treated mice, induces IL-17A response. IECs were co-cultured with pan T cells (a), CD4^+^ T cells (b), and CD8^+^ T cells (c) from control mice in the presence of anti-CD3 antibody. The cytokine concentration in the supernatant was measured by ELISA. The data are representative of four experiments. Results are expressed as means ± standard error (n = 4). ^**^ *p*<0.01 versus co-culture of IECs from control mice and T cells (Control-IEC/T); ^##^ *p*<0.01 versus co-culture of IECs from DSS-treated mice and T cells (DSS-IEC/T). (d) IECs from DSS-treated mice were co-cultured with CD4^+^ T cells in a 96-well plate or Transwell plate. The data are representative of two experiments. Results are expressed as means ± standard error (n = 4). ^**^ *p*<0.01 versus CD4^+^ T cells alone (CD4^+^T); ^##^ *p*<0.01 versus co-culture of IECs from DSS-treated mice and CD4^+^ T cells in a 96-well plate (DSS-IEC/CD4^+^T).

To test if inflamed IECs from colitic mice were involved in the abnormal cytokine profile in the colon, IECs were co-cultured with splenic T cells from non-treated mice. As shown in Figure 3a, IECs from DSS-treated mice (DSS-IEC) induced a high level of IL-17A production (> 1500 pg/ml) and low levels of IFN-γ and IL-10 (< 300 pg/ml) in T cells. When T cells were fractionated into CD4^+^ and CD8^+^ subpopulations, IECs from DSS-treated mice induced IL-17A production mainly in CD4^+^ T cells (Figure 3b,c). This induction was not observed when the cells were co-cultured in a Transwell system, where contact between IECs and CD4^+^ T cells was interrupted (Figure 3d). Notably, IECs from DSS and JCM 1222^T^-treated mice (DSS+*B*.*l*-IEC) induced significantly diminished levels of IFN-γ and IL-17A from CD4^+^ T cells (Figure 3b). In co-cultures of IECs and CD8^+^ T cells, a marked change in IFN-γ and IL-17A production was not observed.

### *B. longum* subsp. *infantis* JCM 1222^T^ suppresses the expression of costimulatory molecules in IEC

**Figure 4:**
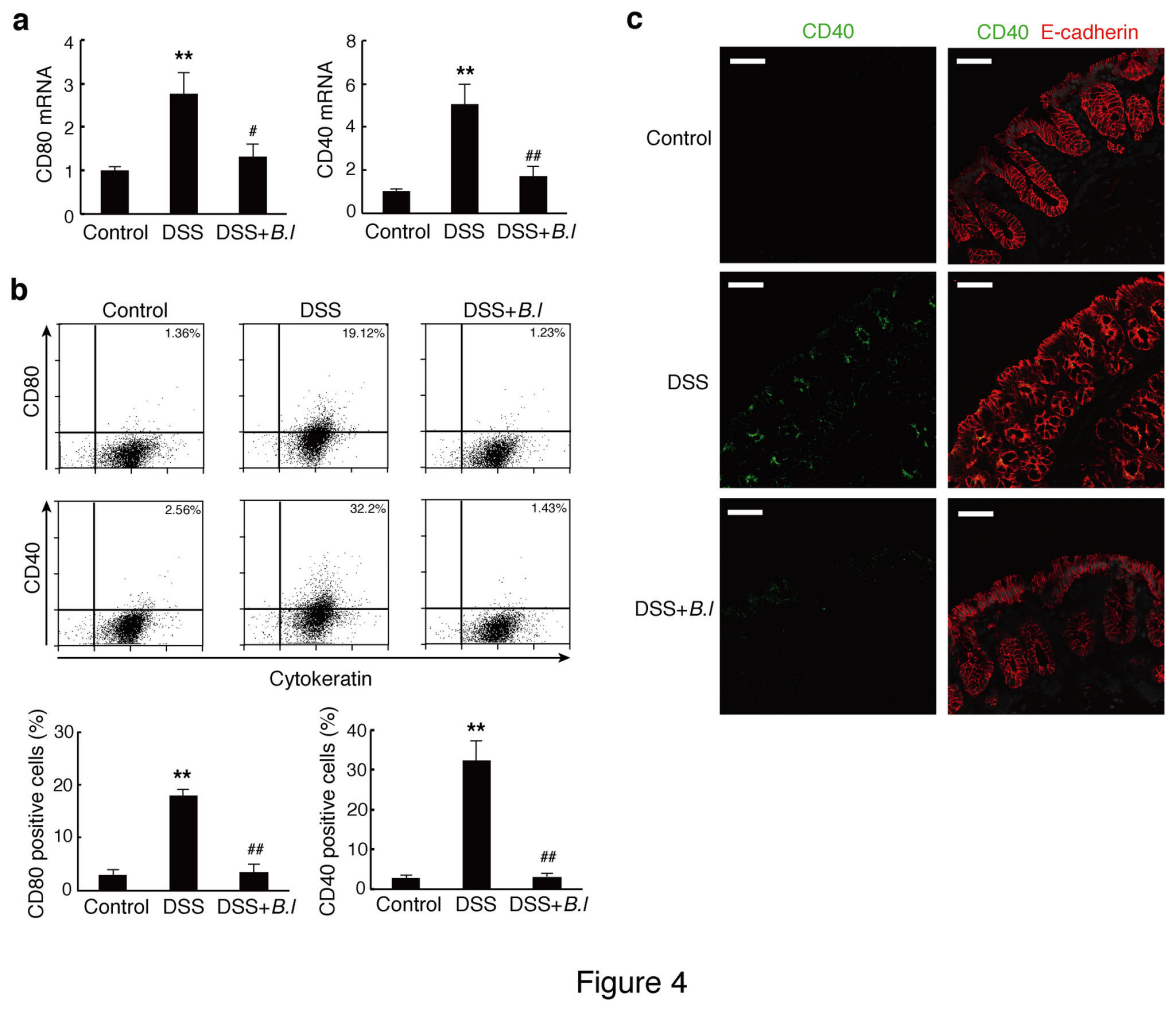
Oral treatment of *B. longum* JCM 1222^T^ (B.l) suppresses the expression of costimulatory molecules in IEC. (a) mRNA expression of costimulatory molecules in IECs was analyzed by quantitative PCR. Levels of mRNA were normalized to β-actin mRNA, and expressed relative to control mice. The data are representative of three experiments. Results are expressed as means ± standard error (n = 6 (Control) or 7 (DSS, and DSS+*B*.*l*)). (b) IECs were stained for cytokeratin and CD80 or CD40 and analyzed by flow cytometry. Debris was gated out by forward and side scatter. Representative plots (upper panel) and the mean and standard error values of the percentage of cytokeratin/CD80 or CD40 positive cells (lower panel) are shown (n = 4). The data are representative of two experiments. (c) Cryosections of colonic tissue were labeled for CD40 (green) and E-cadherin (red). The data are representative of two experiments. Scale bars represent 50 μm. ^**^ *p*<0.01 versus control mice (Control); ^#^ *p*<0.05 and ^##^ *p*<0.01 versus DSS-treated mice (DSS).

Because the recognition of MHC/antigen by the T cell receptor and engagement of costimulatory molecules is required for T cell activation and differentiation, we focused on the expression of these molecules on IEC. mRNA levels of MHC (H-2Ab1 and H-2Eb1) and costimulatory molecules were assessed by quantitative real-time PCR. IECs from DSS mice expressed elevated levels of CD80 and CD40, the most well-studied costimulatory molecules, but not MHC molecules (Figure 4a and Table S1 in File S1). By immunostaining and flow-cytometric analysis, it was revealed that protein levels of CD80 and CD40 were also increased in IECs from DSS mice (Figure 4b,c). However, JCM 1222^T^ oral treatment of DSS mice reduced CD80 and CD40 expression at the mRNA and protein levels (Figure 4a-c). The administration of JCM 1222^T^ alone in control mice did not affect the mRNA expression of these molecules (data not shown).

### Inflamed IEC-induced IL-17A response is CD80/CD86- and CD40-dependent

**Figure 5:**
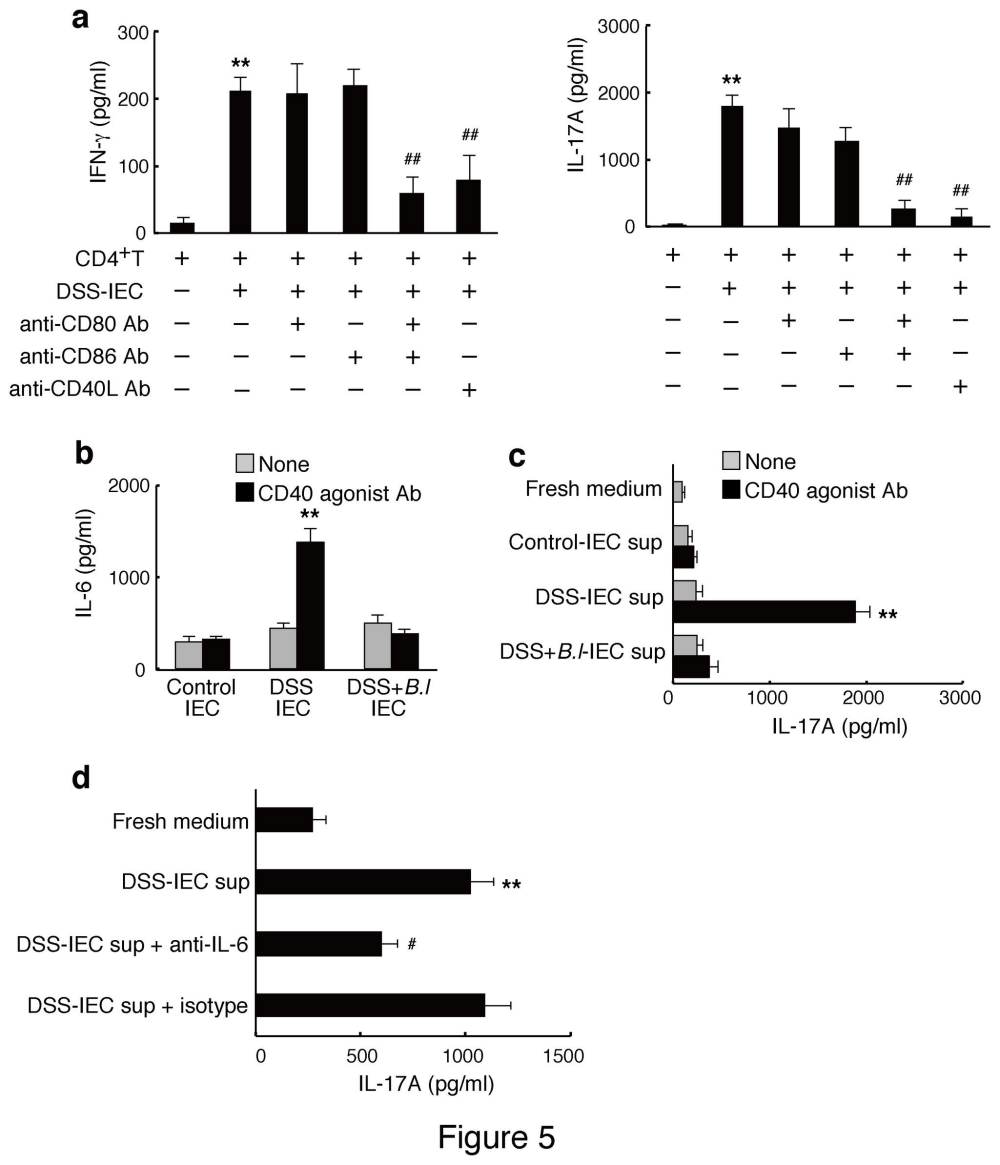
IECs from DSS mice induce IL-17A response via CD80/CD86 and CD40 dependent costimulation. (a) IECs from DSS mice were co-cultured with CD4^+^ T cells from control mice in the presence of anti-CD3 antibody. In the indicated groups, IECs were pretreated with CD80 or CD86 blocking antibodies, and CD4^+^ T cells were pretreated with CD40L blocking antibody before co-culturing. The data are representative of two experiments. Results are expressed as means ± standard error (n = 4). ^**^ *p*<0.01 versus CD4^+^ T cells alone (CD4^+^T); ^##^ *p*<0.01 versus co-culture of CD4^+^ T cells and IECs from DSS-treated mice (DSS-IEC). (b) IECs were cultured in the absence (gray bars) and presence (black bars) of CD40 agonist antibody for 24 h. IL-6 concentration in the supernatant was measured by ELISA. The data are representative of three experiments. (c) CD4^+^ T cells were cultured for 3 days in fresh medium or supernatant from IECs (IECs sup) cultured as described in (b) in the presence of anti-CD3/CD28 antibodies. The IL-17A concentration in the supernatant was measured by ELISA. The data are representative of two experiments. Results are expressed as means ± standard error (n = 4). ^**^ *p*<0.01 versus the supernatant from IECs stimulated with CD40 agonist antibody (CD40 agonist Ab). (d) CD4^+^ T cells were cultured with fresh medium or supernatant from CD40-stumulated IEC of DSS mice (DSS-IEC sup) as described in (c) in the presence of IL-6-neutralizing antibody (anti-IL-6) or IgG control antibody (isotype). The IL-17A concentration in the supernatant was measured by ELISA. Results are expressed as means ± standard error (n = 6). ^**^ *p*<0.01 versus fresh medium; ^#^ *p*<0.05 versus the supernatant from CD40-stumulated IEC of DSS mice (DSS-IEC sup).

To examine whether CD80 and CD40 were involved in inflamed IEC-induction of IL-17A responses, IECs from DSS mice were co-cultured with CD4^+^ T cells in the presence of blocking antibodies for CD80, CD86, or CD40L. IFN-γ and IL-17A production was decreased by inhibition of both CD80 and CD86 (both can ligate CD28 on T cells), but not CD80 alone (Figure 5a). Furthermore, treatment of cells with CD40L blocking antibody alone also resulted in decreased cytokine production. In *ex vivo* cultures, most IECs were dead within 24 h (Figure S1 in File S1) indicating that short-term stimulation (up to 24 h) of CD40 signaling in IECs induced the production of cytokines required for IL-17A secretion. As expected, 24 h stimulation with CD40 agonist antibody induced IL-6 production in IECs from DSS-treated mice (Figure 5b). Moreover, the production of IL-17A, but not IFN-γ (data not shown) was significantly increased when CD4^+^ T cells were cultured in supernatant from CD40-stimulated IECs from DSS-treated mice in the presence of CD3/CD28 antibodies (Figure 5c), whereas increased IL-17A production was not observed in the absence of CD28 antibody (data not shown). The addition of a neutralizing antibody against IL-6 suppressed IL-17A production by CD4^+^ T cells cultured in the conditioned medium from CD40-stimulated IEC of DSS-treated mice (Figure 5d).

### *B. longum* subsp. *infantis* JCM 1222^T^ directly acts on IECs to suppress CD80 and CD40 expression

**Figure 6:**
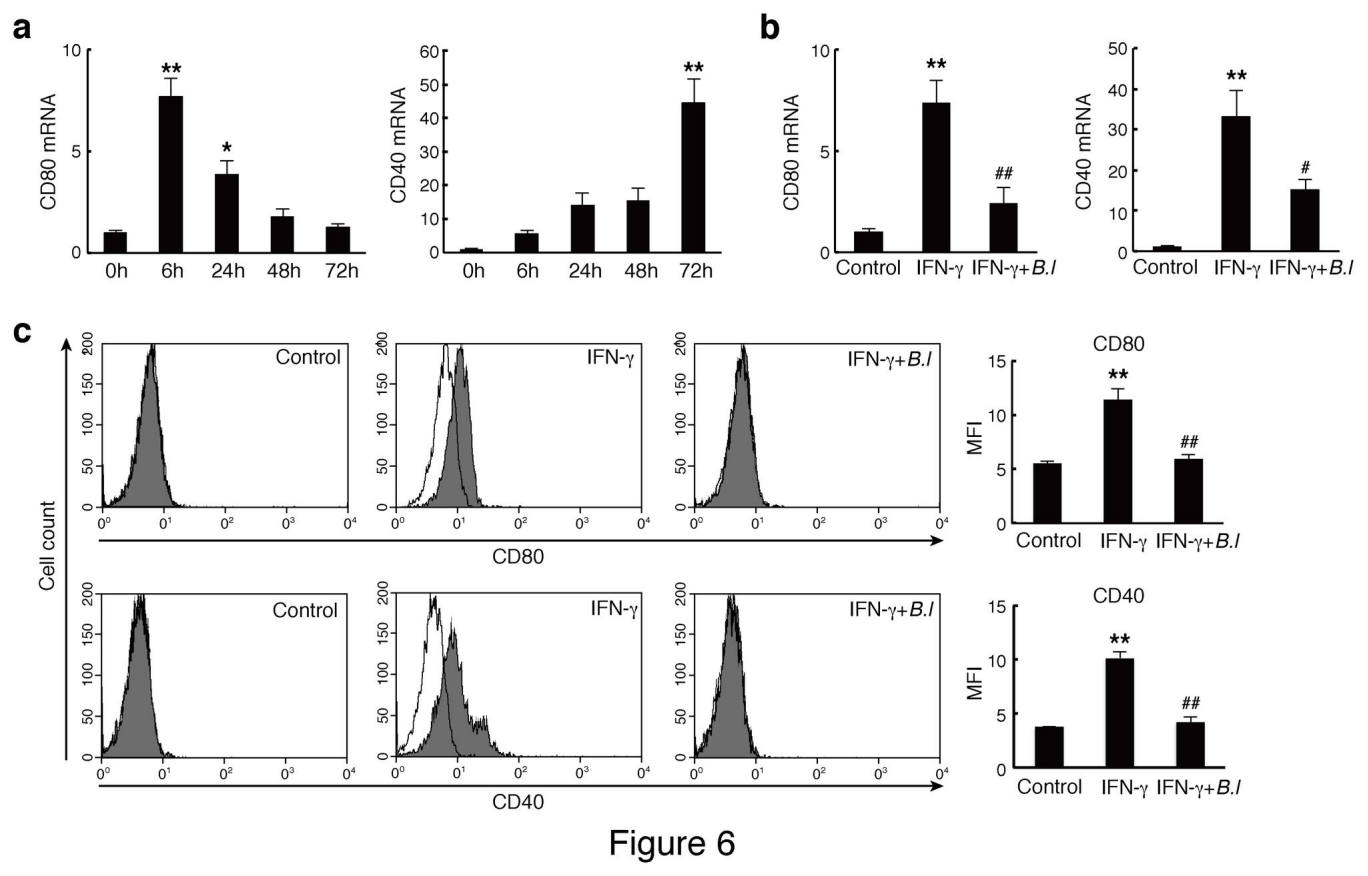
*B. longum* JCM 1222^T^ (B.l) suppresses the expression of costimulatory molecules in Colon-26 cells. (a) mRNA expression of CD80 and CD40 was analyzed by quantitative real-time PCR in Colon-26 cells after stimulation with IFN-γ. Levels of mRNA were normalized to β-actin mRNA, and expressed relative to before stimulation (0 h). Results are expressed as means ± standard error (n = 4). ^*^ *p*<0.05 and ^**^ *p*<0.01 versus before stimulation (0 h). (b) Colon-26 cells were pre-incubated with *B. longum* JCM 1222^T^, and then treated with penicillin and streptomycin and incubated with IFN-γ. Messenger RNA expression of CD80 and CD40 was analyzed after stimulation with IFN-γ for 6 and 72 h, respectively. The data are representative of three experiments. (c) Colon-26 cells were incubated with *B. longum* JCM 1222^T^ and IFN-γ. Cell surface protein expression of CD80 and CD40 was analyzed by flow cytometry after stimulation with IFN-γ. Debris was gated out by forward and side scatter. Histograms show a representative experiment (specific antibody stains are shown in filled histograms, and isotype control antibody stains are shown in open histograms) and bar figures are representative of two experiments. ^**^ *p*<0.01 versus non-treated cells (Control); ^#^ *p*<0.05 and ^##^ *p*<0.01 versus IFN-γ-stimulated cells.

Using mouse intestinal epithelial cell line Colon-26 cells, we examined the direct effects of JCM 1222^T^ on the elevated expression of CD80 and CD40. Both CD80 and CD40 expression was increased at 6 and 72 h after IFN-γ stimulation, respectively (Figure 6a). The protein expression of these molecules was also increased by IFN-γ stimulation (Figure 6c). However, both mRNA and protein expression levels of CD80 and CD40 were suppressed by pre-incubation with JCM 1222^T^ (Figure 6b,c).

## Discussion

This study demonstrated that *B. longum* subsp. *infantis* JCM 1222^T^ alleviated DSS-induced acute colitis via a novel mechanism, by suppressing pro-inflammatory IL-17A response. In addition, it was revealed for the first time that inflamed IECs from DSS-treated colitic mice expressed elevated levels of CD80 and CD40, and induced IL-17A response by cell-cell contact. JCM 1222^T^ suppressed the expression of costimulatory molecules on IECs causing a decreased IL-17A response.

Although DSS-induced acute colitis was originally considered a T cell-independent model, there is evidence that T cell-mediated immunity, especially Th1 and Th17 responses, are involved in the pathogenesis [23,24]. We observed that oral administration of JCM 1222^T^ suppressed the production of Th1- and Th17-cytokines (IFN-γ and IL-17A) and down-regulated transcriptional factors (T-bet and RORγt). Further, IL-17A secreting CD4+ T cells were decreased by JCM 1222^T^, suggesting IL-17A response is the principal target of the anti-colitic effect of this strain.

We also showed that IECs from DSS-induced colitic mice expressed elevated levels of some costimulatory molecules, indicating that IECs could participate in T cell activation and differentiation in DSS-induced colitis. This observation is consistent with other studies showing that IECs from IBD patients abnormally express costimulatory molecules [4-6], and a recent study demonstrating IECs are potential antigen-presenting cells in the colon [25]. In the normal state, IECs suppress CD4^+^ T cell activation and may play a role in the hypo-responsiveness of mucosal T cells [26]. In contrast, inflamed IECs can cause the abnormal activation of T cells by expressing higher levels of costimulatory molecules, and our findings may explain how the hypo-responsiveness of mucosal T cells to luminal antigens is impaired in IBD.

While previous studies revealed that inflamed human IECs stimulate IFN-γ secretion from T cells [4,6], we showed that IECs from colitic mice induced predominantly IL-17A secretion rather than IFN-γ from CD4^+^ T cells. In an *ex vivo* co-culture system, the secretion of IL-17A was inhibited by blockade of CD80 and CD86, but not CD80 alone. IECs constitutively express CD86 [26], and both CD80 and CD86 can bind to CD28 and stimulate T cell activation. Hence, the blockade of CD80 alone may be ineffective to abrogate inflamed IEC-induced T cell activation. Recently, it was shown that patients with IBD, but not healthy controls, expressed functional CD40 in inflamed IEC [5]. In the present study, we also confirmed that IECs from colitic mice expressed functional CD40, and its signaling contributed to IL-6 secretion, a cytokine involved in Th17 differentiation. In dendritic cells (DC), CD40-CD40L ligation induces IL-6 secretion, which leads to Th17 development [27]. The same mechanism may be responsible for the IEC-induced IL-17A response. Indeed, supernatant from CD40-activated inflamed IECs induced IL-17A secretion in CD4^+^ T cells in the presence of CD28 antibody, and this was suppressed by the neutralization of IL-6. However, we cannot conclude that only IL-6 secreted from IECs plays a major role in IL-17A response. Other cytokines such as transforming growth factor-*β* and IL-1β are also critical for Th17 development [28,29]. Further experiments using cytokine-specific blocking antibodies are required to clarify the cytokine(s) involved in IL-17A response induced by IEC. Regarding the interactions between IECs and CD4^+^ T cells, CD4^+^ T cells would be potent inducers of CD40 signaling in IEC. It is well known that CD4^+^ T cells stimulated through CD28 express high levels of CD40L [30]. B7-DC, which is increased in IECs from DSS-induced colitic mice, also induces the elevated expression of CD40L in CD4^+^ T cells [31]. Taken together, our results may highlight a novel aspect of IECs as a IL-17A-inducer in murine colitis, and that costimulatory molecules in IECs such as CD80/CD86 and CD40 may be a therapeutic target for colitis. Further studies using IECs deficient in costimulatory molecules are needed to address this point. 

Here, *in vitro* studies using Colon-26 cells proposed a novel mechanism by which JCM 1222^T^ suppressed colonic IL-17A response. Pretreatment with JCM 1222^T^ effectively suppressed IFN-γ induced CD80 and CD40 expression in Colon-26 cells, implying that this strain may suppress colonic IL-17A response by impacting directly on IEC, not only on immunocompetent cells such as DC. It remains unknown which components or metabolites of JCM 1222^T^ are responsible for the suppression of IL-17A response. Ghadimi et al. reported that DNA from *B. longum* suppressed the activation of epithelial nuclear factor κB (NF-κB) and p38 MAP kinase pathway via toll-like receptor 9 [32]. Other studies demonstrated that secreted components of some bifidobacterial strains exerted anti-inflammatory activity by modulating various cellular signaling pathways in IEC [33-35]. Moreover, we cannot exclude the possibility that JCM 1222^T^ exert an effect by modulating intestinal microbiota, as reported for other *Bifidobacterium* strains [36-38]. Additional studies are required to elucidate the precise mechanisms of JCM 1222^T^, including identifying the effector molecules and the cellular signaling involved in the modulation of costimulatory molecules expression in IEC. Investigation of the relationship between luminal bacteria and epithelial costimulatory molecules may help understand the role of bacteria in IBD. 

In conclusion, this study demonstrated that JCM 1222^T^ suppressed intestinal IL-17A response in DSS-induced colitis. JCM 1222^T^ also regulated the expression of epithelial costimulatory molecules which were involved in the induction of IL-17A secreting CD4^+^ cells. Although other IL-17A producing cells, including γδT cells and innate lymphoid cells, may also be involved in IECs-induced IL-17A response in the colon, these results indicate that epithelial costimulatory molecules could be potential therapeutic targets for Th17-mediated disorders such as IBD. The novel anti-inflammatory mechanisms demonstrated will hopefully lead to the identification and selection of beneficial probiotic strains, and to the improvement of probiotic therapy for IBD [39].

## Methods

### Bacterium

*B. longum* subsp. *infantis* JCM 1222^T^ was obtained from the Japan Collection of Microorganisms (Saitama, Japan). This strain was cultured in GAM broth (Nissui Pharmaceuticals, Tokyo, Japan) under anaerobic conditions using AnaeroPack (Mitsubishi Gas Chemical, Tokyo, Japan) at 37°C for 16 h. The harvested bacterial cells were washed twice with phosphate buffered saline (PBS) and resuspended in PBS at a concentration of 1 × 10^8^ colony forming units (cfu)/ml. The suspensions were stored at −80°C until used.

### Animal studies

BALB/c mice (female, 6-week-old) were purchased from Charles River Japan (Kanagawa, Japan), and all experimental protocols involving animals were approved by the Animal Care Committee, Graduate School of Biosphere Science, Hiroshima University (Permit Number: C10-17). Acute colitis was induced in mice by adding 3.5% (wt/vol) DSS (molecular weight, 36,000–50,000; MP Biomedicals, Aurora, OH, USA) to their drinking water for 5 days. To assess the preventive effects of JCM 1222^T^, mice were fed daily with a 100 μl suspension (1 × 10^7^ cfu) of the bacteria during DSS treatment. DAI was evaluated daily according to the weight loss, stool consistency, and gross rectal bleeding (Table S2 in File S1), as described by Azuma et al. [40]. Colon length was measured as an indirect marker of inflammation. For histological analysis, paraffin sections (5 μm) of colonic tissue were stained by hematoxylin-eosin.

### Colon tissue culture

The distal colon segment (5 cm) was opened longitudinally and washed with ice-cold PBS containing 100 IU/ml penicillin (Life Technologies, Foster City, CA, USA) and 100 μg/ml streptomycin (Life Technologies). The tissue was cut into small pieces (5 mm) and placed into 24-well plates containing RPMI 1640 medium (Life Technologies) supplemented with 10% fetal bovine serum (FBS, ICN Biomedicals, Osaka, Japan), 10 μM 2-mercaptoethanol, 10 mM HEPES, 100 IU/ml penicillin, and 5 μg/ml streptomycin. After 24 h incubation at 37°C in 5% CO_2_, IFN-γ, IL-17A, IL-4, and IL-10 concentrations in the supernatants were measured using DuoSet Sandwich ELISA Kits (R&D Systems, Minneapolis, MN, USA) following the manufacturer’s instructions. The protein concentration of the tissue was measured using Bio-Rad DC Protein Assay Kit (Bio-Rad, Hercules, CA, USA). The concentration of cytokines was expressed as pg/mg protein of colon tissue.

### Isolation of IEC, lamina propria lymphocytes, and T cells

The distal colon segment (5 cm) was opened longitudinally washed with ice-cold PBS and incubated in 1 mM DTT/PBS (Wako Pure Chemical, Osaka, Japan) at room temperature for 10 min under gentle agitation to remove the mucin layer. IECs were isolated by incubation with 30 mM EDTA/PBS at 37°C for 10 min followed by vigorous vortexing for 1 min, and the remaining tissue was used to isolate lamina propria lymphocytes (LPL). Harvested IECs were treated with 2 mg/ml dispase (Life Technologies) and 50 mg/ml DNase I/PBS (Roche Applied Science, Mannheim, Germany) at 37°C for 30 min (vortexing every 2 min). This process was repeated in fresh dispase/DNase I/PBS at 37°C for 30 min. The resultant single IECs were purified by centrifugation through a 20/40% Percoll gradient (GE Healthcare, Little Chalfont, UK). IECs viability (>85%) and purity (>90%) were assessed by trypan blue exclusion and flow cytometric analysis using anti-pancytokeratin (Sigma-Aldrich, St. Louis, MO, USA) and anti-CD45 antibodies (eBioscience, San Diego, CA, USA), respectively.

Colonic tissue without IECs was cut into small pieces (1 mm) and digested in RPMI medium containing 0.5 mg/ml collagenase D (Roche Applied Science), 3 mg/ml dispase, and 50 mg/ml DNase I at 37°C for 1 h with gentle agitation. LPL were purified by centrifugation through a 40/80% Percoll gradient, and only live cells were collected by Dead Cell Removal kit (Miltenyi Biotec, Auburn, CA, USA). 

Splenic T cells (CD11b^-^, CD11c^-^, CD19^-^, B220^-^, CD49b^-^, CD105^-^, MHC-class II^-^, Ter-119^-^) were isolated from non-treated mice and enriched using pan T cell MACS negative selection beads (Miltenyi Biotec), and CD4^+^ and CD8^+^ T cells were subsequently prepared using anti-CD4 Microbeads (Miltenyi Biotec) according to the manufacturer's instructions.

### IEC-T cell co-culture

A total of 2 × 10^5^ IECs were co-cultured with 1 × 10^6^ T cells in 96-well plates containing RPMI complete medium and soluble anti-CD3 antibody (5 μg/ml; Miltenyi Biotec). In some experiments, IECs and CD4^+^ T cells were pretreated with anti-CD40L blocking antibody (clone MR1; Abcam, Cambridge, UK), anti-CD80 blocking antibody (clone 1G10; Abcam) and/or anti-CD86 blocking antibody (clone GL1; Abcam) at 37°C for 30 min before co-culture, respectively. Alternatively, the cells were co-cultured in a 96-well Transwell plate to avoid direct cell contact. IECs and CD4^+^ T cells were seeded in the apical and basal chambers, respectively. After 5 days incubation at 37°C in 5% CO_2_, IFN-γ, IL-17A, IL-4, and IL-10 concentration in the supernatants were measured using DuoSet Sandwich ELISA Kits (R&D Systems). In some experiments, IECs were stimulated with CD40 agonist antibody (Santa Cruz Biotechnology, Santa Cruz, CA) for 24 h, and the supernatants were collected. CD4^+^ T cells cultured for 3 days in the IECs-supernatant with IL-6 neutralizing antibody or species- and isotype-matched antibody (BioLegend, San Diego, CA). The viability of IECs in *ex vivo* cultures was assessed by calcein/propidium iodide uptake [41].

### *In vitro* studies

The mouse adenocarcinoma cell line Colon-26 [42] was provided by RIKEN BRC through the National Bio-Resource Project of MEXT, Japan. The cells were grown in DMEM medium with 10% FBS, 1% nonessential amino acids, and antibiotics (100 IU/ml penicillin, and 5 μg/ml streptomycin) at 37°C in 5% CO_2_. The cells (5 × 10^4^ cells/well) were cultured in 24-well plates containing DMEM medium without antibiotics overnight and pretreated with JCM 1222^T^ (1 × 10^5^ cfu/well) for 2 h. Then, the bacterial growth was stopped by adding penicillin and streptomycin, and the cells were stimulated with 20 ng/ml IFN-γ (Miltenyi Biotec). 

### Quantitative real-time PCR

The total RNA of IEC, LPL, or co-cultured IECs and CD4^+^ T cells was isolated using RNeasy Mini Kit (Qiagen, Maryland, MD, USA), and reverse-transcribed with Verso cDNA Synthesis Kit (Thermo Fisher Scientific, Waltham, MA, USA) at 42°C for 55 min followed by 95°C for 2 min. For Colon-26 cells, Trizol reagents (Life technologies) and High-Capacity cDNA Reverse Transcription Kit (Life Technologies) were used to isolate total RNA and synthesize cDNA (25°C for 10 min and 37°C for 120 min), respectively. Quantitative real-time PCR was performed using an ABI PRISM 7700 Sequence Detection System (Life technologies) and KAPA SYBR FAST qPCR kit (KAPA BIOSYSTEMS, Woburn, MA, USA). The primer sequences used for PCR are shown in Table S3 in File S1. Data were analyzed by the ΔΔCt method [43] and presented as fold changes in gene expression after normalization to the internal control β-actin gene expression level.

### Immunostaining

Frozen sections of colonic tissue were fixed with cold acetone for 10 min. The sections were blocked in 5% normal goat serum and incubated for 1 h with rabbit anti-CD40 antibody (Santa Cruz Biotechnology) and goat anti-E-cadherin (R&D Systems), followed by incubation for 1 h with AlexaFluor 488-conjugated anti-rabbit IgG and AlexaFluor 555-conjugated anti-goat IgG. The specimens were preserved in mounting medium and imaged using a Leica SP5 confocal microscope.

### Flow cytometric analysis

LPL were incubated at 37°C for 5 h in RPMI 1640 medium containing 0.5 μg/ml phorbol-12-myristate-13-acetate (PMA; MP Biomedicals), 1 μg/ml ionomycin (Wako Pure Chemical Industries), and 3 μg/ml brefeldin-A (Wako Pure Chemical Industries). The cells were then blocked with anti-CD16/32 antibody followed by staining with FITC-anti-CD3e and Alexa700-anti-CD4 (BioLegend) at 4°C for 30 min. Intracellular cytokine staining was performed using Cytofix/Cytoperm solution (BD Pharmingen, San Diego, CA, USA), and the cells were stained with APC-anti-IFN-γ and PE-anti-IL-17A antibodies. IECs, and Colon-26 cells were stained with anti-CD80-PE/Cy5 and anti-CD40-APC antibodies at 4°C for 30 min. For cytokeratin staining, the cells were fixed/permeabilized with Cytofix/Cytoperm solution (BD Pharmingen) and then stained with anti-pancytokeratin-FITC antibody at 4°C for 30 min. Species- and isotype-matched antibodies of irrelevant specificity were used as control. All antibodies were obtained from eBioscience unless specified otherwise Flow cytometry was performed using a Guava Easycyte flow cytometer running Guava ExpressPlus software (Merck, Darmstadt, Germany).

### Statistics

All data are expressed as the means ± standard errors. Statistical analysis was performed using one-way ANOVA followed by Tukey’s post-hoc test. A value of *P* < 0.05 was considered significant.

## Supporting Information

File S1 **Supporting Files.** **Figure S1. Viability of IEC in the *exvivo* culture.** IEC from DSS-treated mice were cultured in 96-well plate containing RPMI complete medium. The cells were stained with calcein and propidium iodide at each time point (a), and the viability were evaluated (b). Results are expressed as means ± standard error (n = 3). **Table S1. The expression of costimulatory molecules in IEC from DSS- and DSS+*B*. *Longum* JCM 1222T (B.l)-treated mice.** Levels of mRNA were normalized to β-actin mRNA, and expressed relative to control mice. Results are expressed as means ± SE (n = 6-7). *P < 0.05 versus control mice (Control), and #P < 0.05 versus DSS-treated mice (DSS). **Table S2. Disease activity index scoring.** Clinical criteria were used to evaluate grade of extent of intestinal inflammation. The score ranges from 0 to 12 (total score), which represents the sum of scores from 0 to 4 for weight loss, rectal bleeding, and stool consistency. **Table S3. Primer sequences used in this study.**
(PDF)
