# Supplementary material for: Bifidobacterium longum Alleviates Dextran Sulfate Sodium-Induced Colitis by Suppressing IL-17A Response: Involvement of Intestinal Epithelial Costimulatory Molecules
Source: PLoS One. 2013 Nov 8;8(11):e79735. doi: 10.1371/journal.pone.0079735 (PMC3821848; doi:10.1371/journal.pone.0079735)
Supplement: File S1 — Supporting Files. Figure S1. Viability of IEC in the exvivo culture. IEC from DSS-treated mice were cultured in 96-well plate containing RPMI complete medium. The cells were stained with calcein and propidium iodide at each time point (a), and the viability were evaluated (b). Results are expressed as means ± standard error (n = 3). Table S1. The expression of costimulatory molecules in IEC from DSS- and DSS+B. Longum JCM 1222T (B.l)-treated mice. Levels of mRNA were normalized to β-actin mRNA, and expressed relative to control mice. Results are expressed as means ± SE (n = 6-7). *P < 0.05 versus control mice (Control), and #P < 0.05 versus DSS-treated mice (DSS). Table S2. Disease activity index scoring. Clinical criteria were used to evaluate grade of extent of intestinal inflammation. The score ranges from 0 to 12 (total score), which represents the sum of scores from 0 to 4 for weight loss, rectal bleeding, and stool consistency. Table S3. Primer sequences used in this study. (PDF) [file pone.0079735.s001.pdf]

Supplemental Information

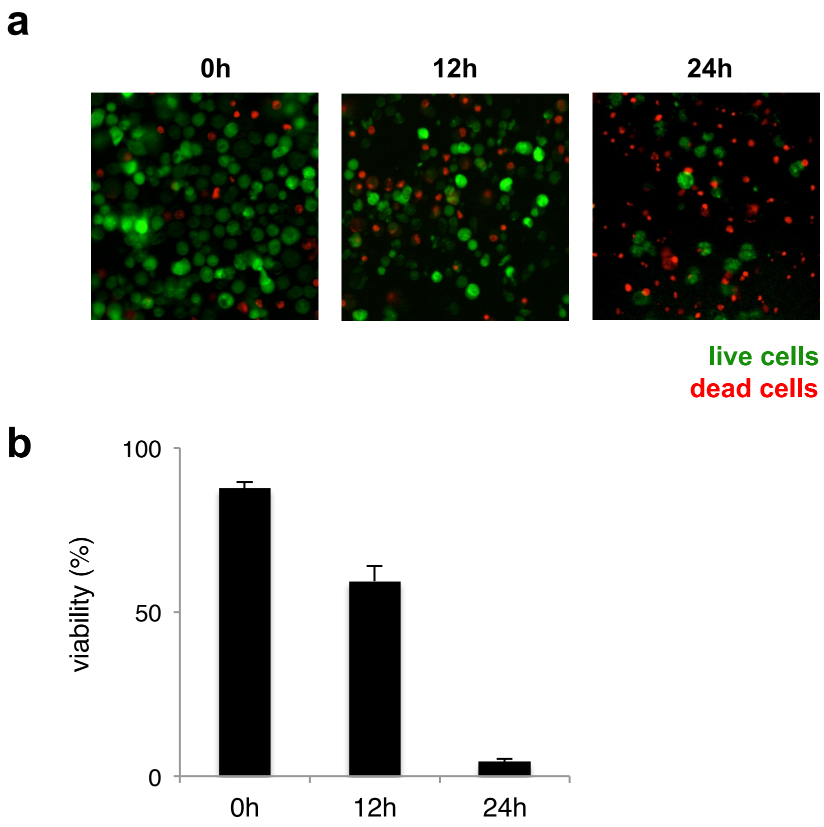

Figure S1. Viability of IEC in the *ex vivo* culture
IEC from DSS-treated mice were cultured in 96-well plate containing RPMI complete medium. The cells were stained with calcein and propidium iodide at each time point (a), and the viability were evaluated (b). Results are expressed as means ± standard error (n = 3).

Table S1. The expression of costimulatory molecules in IEC from DSS- and DSS+*B. longum* JCM 1222^{T} (*B.l*)-treated mice.

	Control	DSS	DSS+*B.l*
CD86	1.00±0.11	1.14±0.10	1.10±0.25
CD54	1.00±0.21	2.20±0.36*	0.97±0.17$^{\#}$
B7h	1.00±0.21	0.78±0.08	0.80±0.21
B7-DC	1.00±0.12	2.38±0.39*	1.90±0.45
B7-H1	1.00±0.29	1.38±0.28	2.10±0.40
H-2Ab1	1.00±0.20	0.95±0.21	0.87±0.26
H-2Eb1	1.00±0.28	1.26±0.22	1.30±0.30

Levels of mRNA were normalized to β-actin RNA, and expressed relative to control mice. Results are expressed as means ± SE (n = 6-7). *P < 0.05 versus control mice (Control), and $^{\#}P$ < 0.05 versus DSS-treated mice (DSS).

Table S2. Disease activity index scoring

Score	Weight loss	Rectal bleeding	Stool consistency
0	None	Normal	Normal
1	1-5%		
2	5-10%	Slight bleeding	Loose stools
3	10-20%		
4	>20%	Gross bleeding	Watery diarrhea

Clinical criteria were used to evaluate grade of extent of intestinal inflammation. The score ranges from 0 to 12 (total score), which represents the sum of scores from 0 to 4 for weight loss, rectal bleeding, and stool consistency.

Table S3. Primer sequences used in this study.

Gene	(Accession No.)		Sequence
T-bet	(NM_019507)	Forword	5'-ACCAGAACGCAGAGATCACTCA-3'
		Reverse	5'-CAAAGTTCTCCCGGAATCCTT-3'
RORγt	(NM_011281)	Forword	5'-AAGCTGAAGGCAGAGACAGC-3'
		Reverse	5'-TGTTCTGGTTCCCCAAGTTC-3'
GATA-3	(NM_008091)	Forword	5'-GTCATCCCTGAGCCACATCT-3'
		Reverse	5'-AGGGCTCTGCCTCTCTAACC-3'
Foxp3	(DQ387959)	Forword	5'-CTGGGTTTAAGATCCCAGCA-3
		Reverse	5'-GAGAGGCCTAGAGCCCTGAT-3
CD80	(NM_009855)	Forword	5'-GCCTTGCCGTTACAACTCTC-3'
		Reverse	5'-TTCCCAGCAATGACAGACAG-3'
CD86	(NM_019388)	Forword	5'-GGGTGGAAGAAAGGTAAAGC-3'
		Reverse	5'-AAGGAAATGAGAGAGACAGGTG-3'
CD40	(NM_011611)	Forword	5'-GGCTTCGGGTTAAGAAGGAG-3'
		Reverse	5'- CCAAAGCCAGGGATACAGG-3'
CD54	(NM_010493)	Forword	5'-TTCCAGCTACCATCCCAAAG-3'
		Reverse	5'-CTTCAGAGGCAGGAAACAGG-3'
B7h	(NM_015790)	Forword	5'-GTTGGCAGCTACAGCAAACA-3'
		Reverse	5'-GGCATCAGGGAGACCACTAA-3'
B7-H1	(NM_021893)	Forword	5'-TCTCCTTTGGGAATGTCAGC-3'
		Reverse	5'-CTGTCAAGGGCTCACACAGA-3'
B7-DC	(NM_021396)	Forword	5'-TTGGGAGTTGGAAGGATCTG-3'
		Reverse	5'-TCCAAAGGGTTCAAAAGTGC-3'
H-2Ab1	(BC008168)	Forword	5'-TCCGTCACAGGAGTCAGAAA-3'
		Reverse	5'-TTCGGAGCAGAGACATTCAG-3'
H-2Eb1	(BC132165)	Forword	5'-CCTGGTCTGCTCTGTGAGTG-3'
		Reverse	5'-TCCTGTTTTCTCCTCCTTGC-3'

β-actin	(NM_007393)	Forword	5'-GATTACTGCTCTGGCCTAGC-3'
		Reverse	5'-GACTCATCGTACTCCTGCTTGC-3'
